# What Was It?

**Published:** 1886-08

**Authors:** J. W. Russey

**Affiliations:** Rising Fawn, Ga.


### WHAT WAS IT?

RISING FAWN, GA., June 9, 1886.

*Editors Atlanta Medical and Surgical Journal:*

I report the following case, as it is a rare one, judging from seeing no mention of a similar one in any work I have ever seen. Ed. Williams, a colored convict, was received at the convict camp at this place, January 5th, 1886, just from jail in Columbus. He was ill at the time of his arrival, complaining of pain around the umbilicus, attended with fever and diarrhœa. He was free from these symptoms but a few days at the time till February 26th, when he failed rapidly, having œdema of feet and legs; bowels distended and tympanitic, with a marked tendency to general anasarca.

About this time intense pain was complained of in left hip, which was relieved by blisters and opiates. There was no swelling, and nothing to denote inflammation in joint. The diarrhœa, which had been previously easily checked, now became uncontrollable.

I recognized the fact that the trouble was entirely abdominal, and diagnosed scorbutus, as I had seen cases of that disease attended with like symptoms in the Tennessee penitentiary, and had decided in my own mind what the autopsy would reveal. He died May 25th of exhaustion. A partial autopsy only was obtained. The abdominal organs only were examined. Incision from ensiform cartilage to symphysis pubis bisected by incision from one flank to the other.

The first abnormal appearance was absence of abdominal cavity. The intestines were matted together and everywhere adherent to the abdominal wall and liver. They were enormously distended with flatus, which had caused the enlargement of abdomen during life. The adhesion of intestines to the parieties was very close, and required considerable force to separate them, and yielded a distinct tearing sound. The bond of union resembled flour paste, and looked very much like that article when only half dry.

The liver was also closely bound to its surroundings, and only its position showed what it was. Its dimensions were as follows: eight inches long, four wide and from three-fourths to an inch thick In color it was a pale pink, everywhere studded with waxy-looking granulations, varying in size from half a grain of wheat to a split bean.

The question is, what was this man's disease? Evidently it was inflammatory in origin, but its cause is not apparent. He did not refer to any antecedent disease as a starting point of his last illness. There was no cough till near the end. His temperature was regular from date of his admission to hospital, viz., 100° to 101.5°, morning and evening respectively. I should very much liked to have made a complete autopsy. From what I saw I am surprised that life could last so long.

Very truly yours, J. W. RUSSEY, M. D.

